# FAILURE TO RECOVER FROM PROACTIVE INTERFERENCE & TRAJECTORY OF CLINICAL DEMENTIA RATING SUM OF BOXES IN AMNESTIC MCI

**DOI:** 10.1093/geroni/igad104.3345

**Published:** 2023-12-21

**Authors:** D Diane Zheng, Rosie Curiel, Alexandra Ortega, Elizabeth Crocco, Kiresten Crenshaw, Sofia Ramirez, Juliana Ortiz, David Loewenstein

**Affiliations:** University of Miami, Miami, Florida, United States; University of Miami Miller School of Medicine, Miami, Florida, United States; University of Miami Miller School of Medicine, Miami, Florida, United States; University of Miami Miller School of Medicine, Miami, Florida, United States; University of Miami, Miami, Florida, United States; University of Miami, Miami, Florida, United States; University of Miami, Miami, Florida, United States; University of Miami Miller School of Medicine, Miami, Florida, United States

## Abstract

**Background:**

The Loewenstein-Acevedo Scale for Semantic Interferences and Learning (LASSI-L) has been shown to be sensitive to Alzheimer’s Disease (AD) pathology among those with preclinical AD, however, LASSI-L’s ability to predict longitudinal cognitive and functional decline of preclinical AD has not been examined.

**Method:**

We evaluated 97 older adults aged 54-98 who were diagnosed with amnestic mild cognitive impairment (aMCI) at 1Florida Alzheimer’s Disease Research Center initial visit. The mean age was 71.9 yr, 51% male, average education 15.7 yr, and mean MMSE score was 28.0. Participants had 3 to 5 visits over a follow-up time as long as 76.7 months. We examined the association of LASSI-L measures on the growth curve trajectory of Clinical Dementia Rating sum of Box (CDRSOB).

**Results:**

The growth curve model that best fit the CDR sum of box trajectory is a linear form and included the fixed and random effect of intercept and slope of time. After adjusting for age, gender, education, Hopkins Verbal Learning Test (HVLT) delayed recall and LASSI-L maximum storage capacity, worse performance on LASSI-L measures of vulnerability to proactive semantic interference (B2 cued recall, β=-0.093, se 0.004, p-0.004) and semantic intrusion (B2 cued intrusions, β=0.053, se 0.023, p=0.02) were statistically significant in predicting a steeper slope on the trajectory of decline in CDRSOB.

**Conclusions:**

The LASSI-L measures reflecting failure to recover from proactive interference and associated intrusions predicted the rate of cognitive/functional decline over time in amnestic MCI and demonstrated the utility of LASSI-L’s longitudinal prediction of prodromal AD.

